# Role of dietary quality and diversity on overweight and obesity among women of reproductive age in Tanzania

**DOI:** 10.1371/journal.pone.0266344

**Published:** 2022-04-07

**Authors:** Heavenlight A. Paulo, Dominic Mosha, Mary Mwanyika-Sando, Innocent B. Mboya, Isabel Madzorera, Japhet Killewo, Germana H. Leyna, Sia E. Msuya, Wafaie W. Fawzi

**Affiliations:** 1 Department of Epidemiology and Biostatistics, Muhimbili University of Health and Allied Sciences, Dar es Salaam, Tanzania; 2 Institute of Public Health, Department of Epidemiology and Biostatistics, Kilimanjaro Christian Medical University College, Kilimanjaro, Tanzania; 3 Ifakara Health Institute, Dar es Salaam, Tanzania; 4 Africa Academy for Public Health, Dar es Salaam, Tanzania; 5 School of Mathematics, Statistics and Computer Science, University of KwaZulu Natal, Durban, South Africa; 6 Department of Global Health and Population, Harvard TH Chan School of Public Health, Boston, Massachusetts, United States of America; 7 Tanzania Food and Nutrition Centre, Dar es Salaam, Tanzania; 8 Department of Nutrition, Harvard TH Chan School of Public Health, Boston, Massachusetts, United States of America; Faculty of Medicine, SERBIA

## Abstract

This study aimed to examine associations of dietary quality and diversity among reproductive-aged women with overweight and obesity. We conducted a cross-sectional study in the Health and Demographic Surveillance System of the Dar es Salaam Urban Cohort Study (DUCS) in Tanzania. A random sample of 1004 non-pregnant women was selected from the DUCS population database and interviewed about dietary information using the FFQ. Women were aged 30.2 (±8.1) years; 27.8% were overweight and 22.6% were obese. All 1004 women in the study consumed starchy staple foods. Of all the women studied, 10.5%, 1.7% and 3.8% consumed vitamin A rich dark green vegetables, nuts and seeds, and beans and peas, respectively. Compared with women in the lowest quintile of Prime Dietary Quality Score (PDQS), those who were in the highest quintile were significantly less likely to be overweight or obese (Adjusted Prevalence Ratio (APR) = 0.76, 95%CI: 0.62, 0.89) (F for trend = 0.029). Dietary diversity score (DDS) was not significantly associated with overweight and obesity. Risk factors included the highest consumption of animal foods (APR = 2.81, 95% CI: 1.51–3.51) and fast food (APR = 2.57, 95% CI: 1.24–4.34). Consumption of legumes and whole grains was associated with lower risk (APR = 0.59; 95% CI: 0.38–0.2). Dietary quality is an important predictor of overweight and obesity among women of reproductive age. Nutrition interventions may be warranted to support women of reproductive age to enter pregnancy with healthier weight to prevent adverse pregnancy outcomes and future risk of chronic diseases.

## Introduction

Overweight and obesity are growing public health problems globally, affecting more than 1.9 billion people. The prevalence of obesity is more among women than men and has tripled between 1975 and 2016 [1]. The pooled prevalence of overweight and obesity among women in 32 sub-Saharan African countries is 22.6%, ranging from 6.7% in Madagascar to 50.7% in Swaziland [2]. The Tanzania Demographic and Health Survey (TDHS) reported that the prevalence of overweight and obesity among women of reproductive age in the country increased from 21% to 28% between 2010 and 2015, with the prevalence that is higher in urban (42%) compared with rural areas (21%) [3]. A recent national nutrition survey in Tanzania also estimated the prevalence of 31.7% for overweight and 11.5% for obesity among women of reproductive age [4].

Overweight and obesity in women of reproductive age has been associated with devastating health consequences such as the increased risk of poor pregnancy outcomes, including gestational diabetes, pregnancy-induced hypertension, pre-eclampsia, postpartum haemorrhage, premature death, neural tube defects, macrosomia as well as increased risks of caesarean delivery and postpartum morbidity during pregnancy [5–7]. Overweight and obesity have also been associated with increased cardiovascular disease, type 2 diabetes mellitus, and some cancers, for example, endometrial and breast cancer [6, 8].

Lifestyle factors such as low physical activity, poor dietary pattern and quality are common known risk factors for overweight and obesity [9, 10]. Thus, the World Health Organization (WHO) recommends individuals limit energy intake from total fats and free sugars but increase consumption of fruits, vegetables, legumes, whole grains, nuts, and polyunsaturated fats [11]. However, there is growing evidence of dietary and nutrition transition in many urban and rural settings in sub-Saharan Africa due to increased economic development, which accelerates the production of processed foods [12]. The latter is experienced in an era of the green revolution in agriculture that focuses on producing starch foods to reduce global hunger and, hence, increased consumption of refined and processed high caloric foods [13, 14].

There is limited information about the role of dietary quality and diversity and how these parameters are associated with women’s weight status in Tanzania. In 2016 the government of Tanzania developed a national nutrition strategy to promote proper nutrition for women of reproductive age, the National Multisectoral Nutrition Action Plan (NMNAP). NMNAP addresses the burden of diet-related non-communicable diseases [15]. Despite these, the prevalence of overweight and obesity among women of reproductive age is increasing [3]. We conducted a community survey in Dar es Salaam, the main commercial city in Tanzania, and assessed dietary quality and diversity among women of reproductive age. The current study aimed to examine the cross-sectional associations between dietary quality and diversity with overweight and obesity.

## Methods

### Study design and population

The study was cross-sectional and nested within Dar es Salaam Urban Cohort Study (DUCS), a Health and Demographic Surveillance System (established in 2011 to support evidence-based planning by providing accurate and timely data on the health status of the general population). DUCS follows 110,882 individuals living in 21,000 households in Dar es salaam, Tanzania [16].

Women of reproductive age (15–49 years) were recruited from September 2018 to January 2019. A list of households with women of reproductive age was initially pulled from the DUCS registers. A simple random sampling technique using random numbers was applied to select households with women of reproductive age. For households with more than one eligible woman, one was randomly selected using a simple random sampling technique (lottery method). Pregnant women were excluded from the study participation based on self-report and estimates of the last normal menstrual period (LNMP). Household replacement was considered in all households without eligible women.

### Study procedures and follow-up

Trained study research assistants collected information on participants’ socio-demographic and economic characteristics. Data on dietary intake, lifestyle and behavioural characteristics, including alcohol use and smoking, were also collected. We used a food frequency questionnaire (FFQ) which contains 85 foods that are commonly consumed in Tanzania [17]. Respondents were asked to recall foods consumed in the past 30 days. The options for responses in the questions were as follows; never consumed, 1–3 servings per month, 1 serving per week, 2–4 servings per week, 5–6 servings per week, 1 serving per day, 2–3 servings per day, and more than 4 times per day. Servings per day were calculated using the following score: never consumed over past thirty days (0 servings per day), 1–3 servings per month (0.067 servings per day), 1 serving per week (0.143 servings per day), 2–4 servings per week (0.429 servings per day), 5–6 servings per week (0.786 servings per day), 1 serving per day (1 serving per day), 2–3 servings per day (2.5 servings per day), and more than 4 times per day (4.5 servings per day). The scores have been used previously in a similar study conducted at DUCS [18].

#### Dependent/Outcome variable

*Anthropometric measurements*. Maternal anthropometric measurements included the assessment of body height (cm) to the nearest 0.1 cm and weight (kg) to the nearest 10 grams. SECA® stadiometer and calibrated SECA® weighing machine were used.

*Overweight/obesity*. The outcome variable in this study was overweight and obesity obtained by computing BMI given as weight in kilograms (Kg) divided by height in meters (m) squared. BMI categories followed the WHO recommendations as 18.5–24.9 kg/m^2^ (normal), 25.0–29.9 kg/m2 (overweight) and ≥30 kg/m2 obese [19]. We combined women in the overweight and obese groups into one category in order to generate a binary outcome and compare women in the overweight/obese group against women in the normal weight group.

### Dietary quality

Women’s dietary quality was assessed using Prime Dietary Quality Score (PDQS), an index that assesses the consumption of healthy and unhealthy food groups associated with chronic disease [20]. PDQS has been used to assess dietary quality in Tanzania [21]. Foods consumed by women were categorised into 19 foods groups, 14 of which are healthy and seven unhealthy food groups. The healthy food group included dark green leafy vegetables, cruciferous vegetables, vitamin A-rich vegetables including carrots, other vegetables, citrus, other fruits, legumes, nuts and seeds, poultry, fish, eggs, whole grains (maise, roasted or boiled maise and sorghum porridge), low-fat dairy, and liquid oil vegetables. Unhealthy food groups include red meat, processed meat, potatoes, refined grains and baked goods, sugar-sweetened beverages, fried foods eaten away from home, and sweets and ice cream.

Scores were allocated based on the frequency of consumption. For healthy food groups, the scores were 0 points for 0–1 serving per week, 1 point for 2–3 servings per week and 2 points for ≥4 servings per week. For unhealthy food, the scoring was reversed as follows: 0 points for ≥4 servings per week, 1 point for 2–3 servings per week, and 2 points for 0–1 serving per week. The PDQS was computed as the sum of scores across the different food groups. We categorised PDQS into quintiles, namely lowest, low, middle, high and highest quintile.

### Dietary diversity

Dietary diversity was assessed by using Minimum Dietary Diversity for Women (MDD-W) based on the guidelines provided by the Food and Agriculture Organization (FAO). The MDD-W has been validated for micronutrient adequacy in LMIC [22]. Foods consumed by women were classified into 10 groups: dark green leafy vegetables, pulses, starchy staples and grains, eggs, vitamin A-rich vegetables and fruits, other vegetables, other fruit (including fruits juice), nuts and seeds, dairy, and animal flesh foods. One point was given for the intake of one serving/ per day for each food group. The MDD-W was computed as the sum of food groups consumed by each woman. The score for MDD-W ranges from 0 to 10 points. We categorised (MDD-W) score into lowest, low, middle, high and highest quintiles.

#### Other independent variables

Five dietary patterns were derived using exploratory factor analysis, a common method for dietary pattern analysis [23]. The patterns are (i) animal products, (ii) vegetables, (iii) western diet, (iv) fast food, and (v) whole grains and legumes patterns. The dietary patterns with eigenvalues >1 and factor loadings of absolute ≥0.3 were retained.

The vegetable pattern was characterised by intake of dark green vegetables, cruciferous vegetables (cabbages) and other vitamins A rich Vegetables (sweet potato leaves, Cassava leaves), cowpea leaves, pumpkin leaves, spinach, and sweet potato leaves). The fast-food pattern was characterised by a high intake of fried foods (fried bananas, potatoes, crisps). The animal product pattern was characterised by a high intake of red meat (beef, goat, and liver) and processed meat. The western dietary pattern was characterised by a high intake of refined grains (white flour, white rice and white bread), desserts and ice cream. The whole grains and legumes pattern was characterised by a high intake of whole grains and legumes such as beans and peas.

Socio-demographic characteristics included age, parity, food security, marital status, level of education and employment. A wealth index was used to measure socioeconomic status and was created using principal component analysis, which is the standard method of analysing wealth index [24]. The first principal component was used as the wealth index and was based on the following variables: availability of electricity and ownership of generator, water source, television, car, motorcycle, Bajaj (three-wheel vehicle for transporting people), Guta (three-wheel vehicle for transporting goods), refrigerator, and Sofa. The input variables were based on ownership of durable assets, for example, cars, television and motorcycle, infrastructures such as water sources, sanitation facilities and electricity.

### Data management and analysis

Data were cleaned and analysed using STATA version 15 (StataCorp. 2017. Stata Statistical Software: Release 15. College Station, TX: StataCorp LLC.). Mean, median and standard deviation, and interquartile range summarised numeric variables. Categorical variables were summarised using frequencies and percentages.

We used modified Poisson regression model with a robust standard error and log link function estimated risk ratios (RR) and 95% confidence intervals (CI) for the association of diet quality and diversity with overweight and obesity. This model was chosen due to the non-convergence of the log-binomial regression models. Potential confounders were identified based on significant association with the outcome variable in the crude analysis. Potential confounders were identified based on significant association with the outcomes variables in the crude analysis. Confounders identified included age group, wealth index status, type of employment and marital status. A stepwise approach with backward selection was used in variable selection, and variables with a p-value less than 0.1 in the crude analysis were used in the multivariable model.

All analyses were based on a two-tailed significance level at p<0.05. The Akaike Information Criteria (AIC) was used for model selection.

### Ethics statement

This study was conducted according to the guidelines laid down in the Declaration of Helsinki. Ethical approval was granted by the Tanzania National Research Ethics Committee (Number NIMR/HQ/R.8a/Vol. IX/2589). Voluntary written informed consent was obtained from all participants. Confidentiality of the data obtained from participants was strictly secured and maintained.

## Results

### Characteristics of the participants

The study enrolled 1004 women of reproductive age. The mean age was 30.2 (±8.1) years, the majority (60%) had only completed primary education, 54.2% were unemployed and more than half (57.9%) were married or cohabiting with their partners. About 49.9% of the women had at least six members in the household, and 54.1% had two or more children. The overall prevalence of overweight and obesity was 50.4%, with 27.8% overweight and 22.6% obese (Table 1).

**Table 1:** Background characteristics of women of reproductive age in Dar es Salaam, Tanzania (N = 1004).

Variable	n (%)
**Age in years**	
**Mean(**±**SD)**	30.2(±8.1)
15–24	334 (33.3)
25–34	351 (35.0)
35 and above	319 (31.7)
**Household size**	5.4 (±2.5)
**Mean(**±**SD)**	
1–3	170 (16.9)
4–5	333 (33.2)
6 and above	501 (49.9)
**Education level**	
No education	59 (5.9)
Primary	600 (59.8)
Secondary	267 (26.6)
Above secondary education	78 (7.7)
**Employment**	
No employment	546 (54.4)
Informal employment	334 (33.3)
Formal employment	124 (12.4)
**Marital Status (N = 1003)**	
Single	356 (35.4)
Married/cohabiting	581 (57.9)
Divorced/separated/widow	66 (6.6)
**Parity**	
One	461(45.9)
Two and above	543 (54.1)
**Minimum Dietary Diversity for women**	
≥5	105 (10.5)
<5	899 (89.5)
**BMI (Kg/m2)**	
Mean(±SD)	25.8 (±5.8)
Underweight	86 (8.6)
Normal	412 (41.0)
Overweight	279 (27.8)
Obese	227 (22.6)

±SD = ±Standard deviation.

### Daily consumption of foods group by quintile of dietary diversity

Overall, nearly all women (99.8%) reported consuming starch staple foods daily, followed by vitamin A-rich fruits and vegetables (70.3%), other fruits (51.7%), other vegetables (27.2%), and fleshy foods/ meats (10.3%). About 3.8% of women consumed beans and peas, and 2.8% consumed dairy products. Consumption of meat ranged from 1.9% in the lowest quintile of DDS to 87.4% in the highest quintile. About 10.5% of women met FAO recommended level of minimum dietary diversity of five or more food groups (Table 2).

**Table 2:** Distribution of food groups consumed daily by quintile of Dietary Diversity Score (N = 1004).

	Food list		1^st^ quintile	^2nd^ quintile	3^rd^ quintile	4^th^ quintile	5 ^th^ quintile	
		Overall	N = 200	N = 201	N = 201	N = 201	N = 201	
Food group		n (%)	n (%)	n (%)	n (%)	n (%)	n (%)	P-value
**Starchy staples**	*African doughnut (mandazi)*, *maise*, *bread*, *cassava*, *chapati*, *irish potato*, *pilau*, *plantain*, *sweet potato*, *taro*, *ugali*	1002 (99.8)	198 (19.8)	201 (20.1)	201 (20.1)	201 (20.1)	201 (20.1)	0.09
**Beans and peas**	*Bambara nuts*, *beans*, *Mung bean (choroko)*, *chickpea (dengu)*, *cowpea (kunde)*, *pigeon peas (mbaazi)*, *peas*	38 (3.8)	1 (2.6)	4 (10.5)	6 (15.8)	7 (18.4)	20 (52.6)	<0.01
**Nuts and seeds**	*Groundnuts*	17 (1.7)	0 (0.0)	0 (0.0)	4(23.7)	5(29.4)	8 (47.1)	0.01
**Dairy**	*Cow’s milk*	29 (2.8)	0 (0.0)	0 (0.0)	0 (0.0)	0 (0.0)	29(100)	<0.01
**Flesh foods (meats)**	*Beef*, *chicken*, *fish*, *goat*, *liver*, *pork*	103(10.3)	2 (1.9)	0 (0.0)	3 (2.9)	8(7.8)	90(87.4)	<0.01
**Eggs**	*Eggs*	0 (0.0)	0 (0.0)	0 (0.0)	0 (0.0)	0 (0.0)	4(100)	0.03
**Vitamin A rich dark green vegetables**	*Cassava leaves (kisamvu)*, *cowpea leaves (kunde leaves)*, *pumpkin leaves*, *spinach*, *sweet potato leaves*	105(10.5)	0(0.0)	1 (1.0)	14 (13.3)	23(21.9)	67(63.8)	<0.01
**Other vitamins A rich fruits &vegetables**	*Peppers (fresh hoho)*, *mango*, *papaya*, *pumpkin*, *passion fruit*, *passion fruit juice*	706(70.3)	29(4.1)	126(17.9)	159(22.5)	192(28.3)	200(28.3)	<0.01
**Other vegetables**	*Bitter tomato*, *chinese cabbage*, *cabbage*, *eggplant*, *hare lettuce (mchunga)*, *okra*, *tomato*, *green maize*	273(27.2)	6 (2.2)	17(6.2)	23(8.4)	65(23.8)	162(59.3)	<0.01
**Other fruits**	*Avocado*, *baobab*, *cucumber*, *guava jackfruit*, *lemon*, *lime*, *orange*, *peach*, *pineapple*, *plum*, *banana*, *tangerine*, *watermelon*, *other fruit juices*	519(51.7)	17 (3.3)	53 (10.2)	118(22.7)	159(30.6)	172(33.1)	<0.01
**Sugar Sweetened beverages**	*Malts drinks*, *energy drinks*, *coffee or tea with sugar*, *sweetened fruits juices*, *and soft drinks*	8(0.8)	0(0.0)	0(0.0)	0(0.0)	2(25.0)	6(75.0)	0.002
**Fried and Salt foods**	*samosa*, *packed salts snacks*, *fried cassava balls*, *cassava chips*, *potato chips*, *snacks foods*, *and corns*	124 (12.4)	9 (7.3)	21(16.9)	25(20.2)	30(24.2)	39(31.5)	<0.01
**Sweet foods**	*Biscuits*, *cakes*, *ice cream*, *cookies and frozen yoghurt*	11(1.1)	1(19.9)	0(0.0)	2(18.2)	4(36.4)	4(36.4)	0.209

### Healthily and unhealthily foods consumed per week

The dietary quality scores (PDQS) ranged from 3 to 27. Majority (88.8%) consumed 4 or more servings of other vitamin A vegetables per week. About 0.2% consumed ≥4 servings of cruciferous vegetables per week. One-quarter of the women consumed 2 to 3 servings of legumes per week. About 37.5% of women reported consuming 4 or more servings of dark green leafy vegetables per week, and 52.6% consumed 4 or more servings of other vegetables per week. About 0.2% consumed ≥4 servings of cruciferous vegetables per week. One-quarter of the women consumed 2 to 3 servings of legumes per week. Only 0.5% consuming 4 or more serving of eggs per week. The majority of women (80.5%) consumed 4 or more servings of refined grains and baked foods per week, and 95.5% consumed 2 to 3 servings of sugar-sweetened beverages per week (Table 3).

**Table 3:** Prime dietary quality score food groups consumed per week (N = 1004).

Food groups	Servings and points		
	0–1 serving/wk (0 point)	2–3 servings/wk (1 point)	≥4 servings/wk (2 points)
	n (%)	n (%)	n (%)
**Healthy foods**			
Cruciferous vegetables	967 (96.3)	35 (3.4)	2 (0.2)
Dark leafy green vegetables	309 (30.8)	319 (31.8)	376 (37.5)
Eggs	982 (97.8)	17 (1.7)	5 (0.5)
Fish	891 (88.8)	44 (4.4)	69 (6.9)
Legumes	332 (59.9)	515 (25.1)	157(15.6)
Nuts	867(86.4)	98 (9.8)	39 (3.9)
Other vegetables	263 (26.2)	213 (21.2)	528 (52.6)
Other vitamin A rich vegetables	8 (0.8)	105 (10.5)	891 (88.8)
Other whole fruits	119 (11.9)	128(12.8)	757 (75.4)
Poultry	987(98.3)	16 (1.6)	1 (0.1)
Whole citrus fruits	257 (25.6)	251 (25.0)	496 (49.4)
Whole grains	143 (14.2)	234 (23.3)	627 (62.5)
**Unhealthy foods score**	**(2 points)**	**(1 point)**	**(0 Point)**
Desserts and ice cream	998 (99.4)	3 (0.3)	3 (0.3)
Fried foods obtained away from home	283 (28.2)	342 (34.1)	379 (37.8)
Potatoes	739 (73.6)	202 (20.1)	63 (6.3)
Processed meat	1000 (99.6)	3 (0.3)	0 (0.1)
Red meats	924 (92.0)	80(8.0)	0 (0.0)
Refined grains and baked goods	27 (2.7)	157 (16.8)	808 (80.5))
Sugar sweetened beverages	45(4.5)	959 (95.5)	0 (0.0)

Abbreviations: Wk: week.

### Dietary quality and diversity and association with overweight and obesity

In the adjusted analysis, the PDQS was significantly associated with a lower risk of overweight/obesity (p for trend = 0.029). Women in the highest and high quintiles of PDQS had a 24% (95%CI: 0.62, 0.89) and 19% (95%CI: 0.66, 0.99) lower risk of overweight/obesity, respectively 0.89) compared to women in the lowest PDQS quintile in this population, p for trend 0.029. The dietary diversity was not significantly associated with overweight and obesity (Table 4).

**Table 4:** Association between dietary quality and diversity with overweight and obesity (N = 918).

Variable	Overweight/obese n (%)	CPR*(95%CI)	P-value	APR^†^(95%CI)	P-value	P-value for trend
**PDQS**						
Lowest	153 (55.6)	1		1		
low	68 (54.8)	1.01 (0.83,1.21)	0.093	0.79 (0.62,1.01)	0.063	
medium	90 (52.0)	0.91 (0.76,1.12)	0.246	0.83 (0.69,0.98)	0.031	0.029
high	113 (55.7)	1.09 (0.91,1.30)	0.345	0.81 (0.66,0.99)	0.043	
highest	82 (57.3)	1.03 (0.85,1.23)	0.787	0.76 (0.62,0.89)	0.028	
**DDS**						
Lowest	100 (54.6)	1		1		
Low	103 (56.0)	1.07 (0.94,1.23)	0.311	1.09 (0.84,1.41)	0.536	
Medium	87 (47.5)	0.92 (0.76,1.12)	0.399	0.88 (0.67,1.16)	0.352	
High	112 (60.9)	1.09 (0.91, 1.30)	0.345	1.15 (0.89,1.49)	0.281	0.241
Highest	104 (56.5)	1.02 (0.85,1.23)	0.787	1.11 (0.86,1.43)	0.429	

*Crude prevalence ratio.

^†^Adjusted prevalence ratio -adjusted for age, wealth index, type of employment, marital status, number of parity, physical activity and education level.

### Dietary patterns and associations with overweight and obesity

In a multivariable-adjusted model for dietary pattern, there was a positive relationship of significant risk increase of overweight and obesity with the increase of animal product tertile. The risk was high in the medium tertile (PR = 1.57, 95%CI: 1.08, 2.28) and highest in the high tertile (PR = 2.81, 95% CI: 1.51, 3.51). A similar trend was also observed in the fast food; women in the highest tertile of fast food consumption had a higher risk of being overweight (PR = 1.34, 95%CI: 1.24, 4.34) compared to women in the lowest tertile of fast food consumption. Women in the highest tertile of intake of whole grains and legumes had a lower risk of overweight and obesity (RR = 0.59, 95%CI: 0.38, 0.92) compared to those in the lowest tertile, in models adjusted for age, wealth index, type of employment, marital status, number of parities, education and physical activity (Table 5).

**Table 5:** Association between dietary patterns with overweight and obesity (N = 918).

Dietary pattern	Overweight/obese n (%)	APR^†^(95%CI)	P-value	P-value for trend
**Animal product**				
Low	167 (54.6.0)	1		
Medium	166 (54.3)	1.57 (1.08,2.28)	0.02	
High	173 (56.3)	2.81 (1.51, 3.51)	<0.01	<0.01
**Vegetables**				
Low	166 (54.3)	1		
Medium	167 (54.6)	0.84 (0.58, 1.22)	0.37	
High	173 (56.5)	1.34 (0.76, 2.37)	0.30	0.23
**Fast food**				
Low	168 (54.9)	1		
Medium	163 (53.3)	2.13 (1.29, 3.53)	<0.01	
High	175 (57.2)	2.57 (1.24, 4.34)	0.01	0.01
**Western diet**				
Low	165 (53.9)	1		
Medium	166 (54.3)	0.99 (0.58, 1.71)	0.98	
High	175 (57.2)	1.32 (0.92,1.91)	0.12	0.38
**Whole grains and legumes**				
Low	168 (54.9)	1		
Medium	170 (55.6)	0.84 (0.58, 0.87)	0.04	
High	168 (54.9)	0.59 (0.38, 0.92)	0.02	0.04

^†^Adjusted prevalence ratio -adjusted for age, wealth index, type of employment, marital status, number of parity, physical activity and education level.

## Discussion

We evaluated the association of dietary diversity and quality with their risk of overweight and obesity. We found that dietary quality was inversely associated with the risk of overweight and obesity in women of reproductive age. Dietary diversity was not significantly associated with overweight and obesity. Starchy staple was the food group consumed by nearly all participants, followed by fruits and vegetables. Dairy products, nuts and seeds were the least consumed food groups.

We found that 10% of women ate at least five groups of DDS. This may be contributed by the food market system observed in many African cities whereby there is a good supply of energy-dense foods. Additionally, the urban lifestyle has favoured increasing vendors selling processed foods rich in calories [12]. Recent studies from Dar es Salaam showed that more than 50% of households purchase snacks and soda for consumption every day [25]. This agrees with our findings on the high consumption of health-risk foods among women of reproductive age.

We found that dietary quality was significantly associated with a low risk of overweight and obesity. Our study findings on the relationship with dietary quality are consistent with previous similar studies in China, USA and Canada, which reported a negative relationship between dietary quality and overweight and obesity [26–28]. Our study also found high consumption of unhealthy foods such as desserts and ice cream, sugar-sweetened beverages, and red meat, with most women consuming at least four servings per week. A systematic review conducted in sub-Saharan countries reported high protein and sugar intake increased the risk of overweight and obesity [29]. Industrialisation in Dar es Salaam and a poor environmental condition characterised by a high temperature may explain the increased consumption of drinks with added sugar [30]. The global shift in diet and an increase in the advertisement of unhealthy foods might have had influenced the consumption of energy-dense foods with low micronutrients and fibres [31].

There was no association between DDS and overweight and obesity. This is consistent with findings from a systematic review and meta-analysis by Akbari *et al*. [32], as well as a previous study in Tanzania [33]. The finding contrasts with studies from Sri Lanka, Iran, and China, which found that DDS is positively associated with an increased risk of overweight and obesity [34–36]. Another study in the Philippines found that dietary diversity was negatively associated with overweight and obesity [37]. The limitations of MDD-W could explain the observed difference as it estimates micronutrient adequacy and not considering overnutrition. Women with more diversified diets may also be consuming more unhealthy food interpreted as poor dietary quality. This may be the case like Dar es Salaam where the present study was conducted, having high accessibility of unhealthy foods [25]. Consumption of nutrition-rich foods was found to be very low, and more than three-quarters of women did not attain minimum dietary diversity.

The DDS and PDQS differed in their associations with the risk of overweight and obesity in this study. The PDQS estimates could explain the difference, which considers the consumption of unhealthy energy-dense foods and scores them negatively. The PDQS measures aspects of diet quality such as energy balance and moderation in consuming unhealthy foods associated with overweight/obesity and chronic diseases [35]. The DDS only assesses dietary diversity and micronutrient adequacy and may not be sensitive to unhealthy diets related to overnutrition.

We found high consumption of animal products significantly associated with overweight and obesity, which is consistent with another study in Tanzania by Keding et al.) that associated consumption of animal fats with increased risk of overweight and obesity through increased consumption of saturated fats and higher energy intake [38, 39]. A systematic review in the USA reported that food sources from an animal product are significantly associated with NCDs [40], a secondary consequence of overweight and obesity. The consumption of red meat in Tanzania is rising very fast, predicted to increase by 71% between 2017 and 2022, and Dar es Salaam is leading as the major meat market in the country [41].

Furthermore, high consumption of fast foods characterised by eating fried foods such as potatoes increases the risk of overweight and obesity. Consumption of fried foods and snacks was associated with higher odds of overweight and obesity in Indonesia [42]. Potatoes are known to be rich in carbohydrates and energy. Fried foods are rich in fats and energy, contributing to overweight and obesity [43, 44]. Findings from a systematic review by Schlesinger et al., reported that whole grains and legumes lower the risk of overweight and obesity [45], similar to our present study findings. These imply that consuming a diet rich in whole grains and legumes can assist in reducing weight gain. Whole grains are rich in fibre and other micronutrients that are essential in health [46].

This study found that the overall prevalence of overweight was 27.5% and obesity was 23.1%. This is higher than the national prevalence of 42% estimated in the urban setting of Tanzania [3] and 48.6% in Dar es salaam that was reported recently in the National Nutrition Survey [4]. The prevalence is also higher than in many sub-Saharan countries such as Uganda (37.2%) and Zimbabwe (36.6%) [30] as well as the pooled prevalence (15.9%) from a systematic review in 32 sub-Saharan countries [2] The observed high prevalence in the study areas could be due to nutrition transition fuelled by urbanisation and industrialisation, as reported in other urban settings [29].

This is one of the few studies in sub-Saharan Africa that managed to demonstrate the role of dietary diversity and quality among women of reproductive age. The study is among the first studies that managed to characterise dietary quality and diversity in sub-Saharan Africa and are related to the risk of overweight and obesity among women of reproductive age. This may provide actual guidance when designing a potential and high impact intervention package for controlling body weight in this particular population. However, the study had some limitations, including recall bias, as some respondents may fail to remember foods consumed in the past 30 days. The observed association between dietary quality and diversity and risk of overweight and obesity in our study should not be interpreted as a cause and effect relationship due to the cross-sectional study design. Finally, this study has been conducted in the urban area, and therefore, the findings may not be generalised to populations in rural areas.

## Conclusion

The combined prevalence of overweight and obesity among women in Tanzania’s leading commercial city was high. The consumptions of energy-dense foods, including red meat and sweet beverages were also high. Improving dietary quality may reduce the risk of overweight and obesity. Our findings call for an urgent nutrition intervention to support women to have healthier weight before pregnancy and prevent immediate and future health consequences, including the risk of adverse pregnancy outcomes, diabetes, and hypertension.
